# Plastic recycling: A panacea or environmental pollution problem

**DOI:** 10.1038/s44296-024-00024-w

**Published:** 2024-08-01

**Authors:** Nisha Singh, Tony R. Walker

**Affiliations:** 1 Japan Agency for Marine-Earth Science and Technology (JAMSTEC), 2-15 Natsushimacho, Yokosuka, Kanagawa 237-0061 Japan; 2 School for Resource and Environmental Studies, Dalhousie University, Halifax, NS B3H 4R2 Canada

**Keywords:** Environmental sciences, Materials science

## Abstract

Increasing plastic waste is a critical global challenge to ecological and human health requiring focused solutions to reduce omnipresent plastic pollution in the environment. While recycling has been touted as one solution to counter plastic waste and resource utilization, it has been largely ineffective in offsetting the impact of rising global plastic production of more than 400 million metric tonnes annually, due to low global recycling rates of only 9%. Over three decades since implementing plastic resin codes, recycling has favoured thermoplastics, neglecting thermoset plastics. There is a constant need to enhance overall recycling efficiency by exploring advanced methods, as enormous gaps exist in fully unlocking the potential of plastic recycling. We identify critical gaps associated with plastic waste recycling and its potential environmental impacts. We discuss substantial progress in recycling technology, designs-for-recyclability with controlled chemical use, and economic incentives to expand markets for recycled plastics and to curb plastic leakage into the environment. Additionally, we highlight some emerging strategies and legally binding international policy instruments, such as the Global Plastics Treaty that require further development to reduce plastic waste and improve plastic recyclability.

## Introduction

The versatile properties of plastics, in contrast to traditional materials such as paper, glass, and metals, facilitate innumerable applications across various sectors, including automobiles, agriculture, electronics, packaging, and healthcare^1,2^. For example, the incorporation of plastic in various vehicle components reduces weight and enhances performance in automobile industries. Our growing reliance on the convenience of consumer plastics has resulted in increased global production and consumption leading to unprecedented plastic waste generation and widespread plastic pollution. However, our infatuation with plastics is weakening due to its associated risks to environmental and human health^3,4^.

**Fig. 1:**
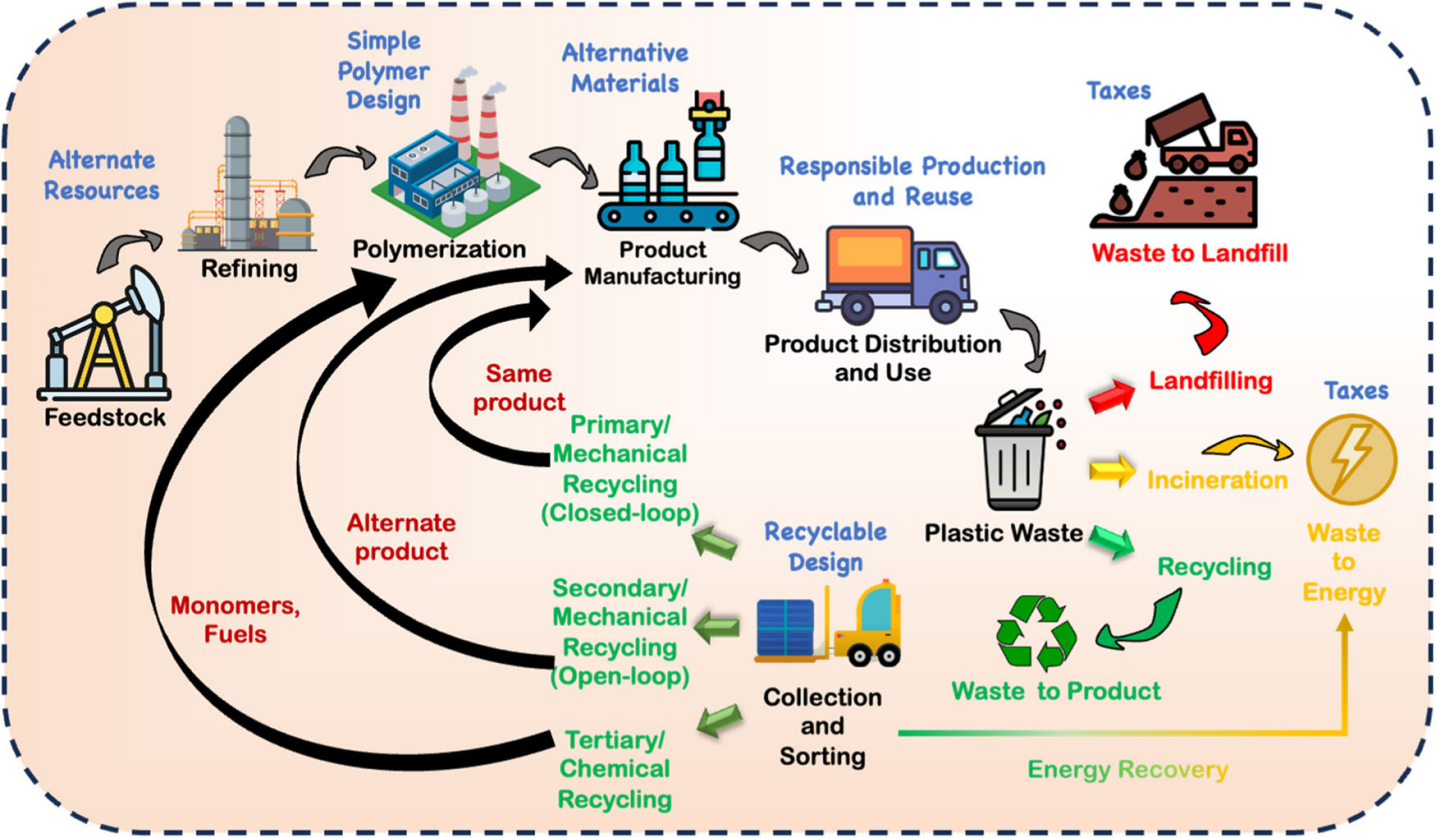
Schematic showing the plastic life cycle (black), different plastic waste handling methods (landfilling, incineration, and recycling), approaches to recycling (green), and solutions to achieve sustainability (blue). Artwork for this figure is original and created by the authors.

Globally, more than 9200 million metric tonnes (Mt) of plastic have been produced to date. Of this, a significant 6900 Mt has not undergone any type of recycling, resulting instead in accumulation in landfills or dispersal within the environment. This represents a missed economic opportunity and a substantial detriment to the environmental health^5^. To sustain the viability of this multi-billion-dollar material, it is crucial to address the complexity of plastic waste and take transformative steps to redesign plastic products focusing on sustainability and end-of-life (EoL). Among the recently available options to manage plastic waste are – (1) landfilling (waste-to-landfill), with its finite capacity, risks leaching toxic chemicals into the surrounding environment, (2) waste-to-energy through incineration with the potential to release hazardous chemicals and gases (e.g., dioxins and furans), and (3) recycling plastic waste into new products^6,7^ (Fig. 1). Plastic waste in landfills is a reflection of unrealized economic potential and harm inflicted upon the environment. While energy recovery from plastics offers convenience without the labour-intensive sorting required for recycling, it limits material recovery to low energy conversion and intensifies atmospheric pollution and global warming. However, emerging carbon capture technologies in exhaust gases may be used so that CO_2_ emissions can be minimized^6^. Conversely, recycling presents an opportunity to address the challenge of increasing global plastic waste.

Plastic recycling encompasses the entire process from waste collection to reprocessing into valuable form^8^ (Fig. 1). Plastics can undergo mechanical or chemical recycling to maintain their original chemical structure, or deliberately alter the chemical composition of the material, respectively^9,10^. Currently, mechanical recycling dominates plastic waste management^11,12^, with polyethylene or polythene (PE) and polyethylene terephthalate (PET) being the most commonly recycled^8,13^ and valuable post-consumer plastics globally. Plastic recycling is performed using different approaches including primary, secondary, tertiary, and quaternary recycling^6,8^. A small fraction of mechanically recycled plastics undergo closed-loop material recycling to generate identical products as the original plastic and contribute to primary recycling. As a result, closed-loop recycling relies on high-quality waste inputs^14^, with pre-consumer manufacturing waste forming a crucial component^6^. Additionally, open-loop recycling creates products serving different purposes than the original material that enters into alternative markets^11^. The process of open-loop mechanical recycling can potentially lead to secondary recycling opportunities. Conversely, tertiary, or chemical cycling practices advance methods to depolymerize and recover monomers, and hydrocarbon products through pyrolysis, and gasification^15^. Chemical recycling, while efficient for mixed plastic waste, is quite limited due to high energy requirements and intense reaction conditions. Besides, a major burden of chemical recycling technologies such as gasification or pyrolysis is the need to clean the downstream output— to protect the equipment and keep the product valuable^16^. Further, the quaternary approach involves energy recovery by incineration, especially from mixed plastic waste instead of diverting it to landfills^6^.

Theoretically, most polymers are recyclable and some even have desirable cradle-to-cradle lifecycles, offering opportunities for a circular plastic economy^12,17^. Here, we discuss some major challenges of recycling such as the complexity of plastic products themselves, market forces that make fossil-fuel-derived virgin plastics cheaper than recycled plastic feedstock, the negative environmental, and social impacts, and inconsistent global policies, including the Global Plastics Treaty^18^, that influence international efforts for effective closed-loop plastic recycling^19^. Additionally, we call for prioritizing reduction in plastic production, consumption, and exploring alternative sustainable materials to tackle rising plastic waste^20,21^.

## Challenges of plastic recycling

Acknowledging the presence of EoL plastics is crucial in addressing the intricacies of plastic recycling. While recycling is widely touted as a promising pathway to achieving a plastic waste-free future, there remain substantial barriers to making this a reality. For example, current global recycling rates, at only 9%, are simply ineffective in the face of increased plastic production. Over 400 Mt of plastics is produced annually^2^, primarily as single-use items, accounting for more than 50% of consumer-based plastics, which are difficult-to-recycle^22,23^. The intrinsic polymer and product design flows of plastic impede their EoL recyclability. Despite the recyclability of most consumed thermoplastics, only a small fraction of them find their way into the recycling stream. Besides, several plastics are incompatible during recycling resulting in a phase-separate mixture adding to the recycling cost and reducing profitability^6^. Meanwhile, the phase separation of the mismatching plastic waste stream can be controlled by polymer compatibilizers such as block copolymers, and graft copolymers^24^. The introduction of compatibilizers stabilizes the immiscible mixture and allows their interaction to produce advanced material^12,13^.

Contemporary recycling techniques predominantly address thermoplastics, omitting a substantial fraction of plastic types lacking circular design. Thermoset plastics exemplify this issue, where their valued rigidity from covalent cross-linking also confers significant recycling resistance^12^. While it is possible to grind into fine powders for certain downgrade applications, recycling thermosets, which currently constitute one-third of the total plastic manufactured, requires a distinct approach compared to thermoplastics^25^. Similarly, elastomers primarily composed of tires, represent one of the rapidly expanding industries, and encounter an uncertain fate^24,26^. Additionally, composite plastics, integrating polymers with fibrous substances such as fiberglass or carbon fibre, are increasingly used across various industries but present substantial separation hurdles. These challenges underscore the imperative for research into the design of easy-to-recycle plastic materials^17,27^.

The complexity and diversity of plastic compositions, exacerbated by chemical additives blended for versatility, lead to a low recycling rate due to the difficulty in recycling different grades together without degrading properties^11^. For instance, reprocessing different colours of 100% recyclable PET^28^ together can lead to lower-quality recyclate^19,24^. High-value transparent plastics are preferred and hold higher market value, while pigmented ones may be discarded. Therefore, recycling necessitates extensive sorting facilities to maintain the quality of the end product. A notable challenge to sorting lies in the complex composition of most plastic waste generated today, compounded by contamination with labels, coatings, and food remains^8,29^. The immiscible plastic waste, combined with diverse materials, questions the efficacy of current recycling techniques, which are more inclined to pure waste polymers requiring efficient waste collection and extensive sorting^24^. Although sorting waste at the source has generally improved, the sorted waste is often underutilized or repurposed ineffectively^30^. If the waste stream is too contaminated, it is not recycled and diverted to landfills or incinerators^19^. Moreover, recycled plastics typically endure only a few recycling cycles^3^, with approximately 10%—undergoing multiple rounds^31^, and are often mixed with virgin materials to maintain the desired properties^24^.

About 90% of plastics production relies on oil and gas feedstocks^23^, and in 2019, this accounted for 6% of the world’s oil production used as raw material^32,33^. The surge in fossil fuel availability for plastic production, driven by global decarbonization efforts in the energy and transport sector, exacerbates the issue. Recent developments in creating alternative materials like bio-PET and bio-PE aim to promote reduction of fossil resource use and to reduce life-cycle CO_2_ emissions. Incorporating these bioplastics, identical to their fossil fuel versions, into existing recycling methods, however, remains crucial to their positive impact and avoid waste problems and plastic pollution at EoL^34^. The readily available and inexpensive fossil fuels present a significant disincentive to building waste collection infrastructure, particularly in low-income countries where funding and planning are already insufficient. This poses substantial challenges to enhancing recycling efforts and developing a more robust waste management system^4,35^.

Consequently, the low recycling rate leads to a disparity between the demand and supply of recycled plastic resins^36^. Additionally, market values of reprocessed resins are compromised by their reduced structural integrity. Advanced techniques, such as solid-state polymerization, offer solutions by enhancing polymer chain reassembly and strength by heating the polymer without reaching melting points. Often contaminated plastic waste from industries or agriculture chemical packaging limits the application of recycled products^1^. The ambition to incorporate more recycled plastics into products confronts the reality of the shortage of high-quality and volume plastic waste and reprocessed resins^37^. Regardless embracing plastic recycling, has the potential to generate substantial profits of up to USD$60 billion by 2030, within the petrochemicals and plastics sector^37^. However, utilizing recyclates as direct replacements for virgin plastics is crucial to undercut the production of the latter and to prevent the proliferation of low-end, disposable goods. A strategic shift in the market towards high-quality recyclable materials is essential for bridging the existing gap in the recycling ecosystem and for the realization of the sector’s financial potential.

Among other challenges to the unique composition of every plastic and availability of cheap virgin plastics, the lack of consistency and standardization in waste handling approaches are major obstacles across the globe. The Resin Identification Code (RIC), is defined for polymers under the 1-6 category, while category 7 includes all others^7^, with no dedicated class for nonrecyclable, biodegradable polymers such as polylactic acid, and elastomers including rubbers. Since the inception of RIC in 1988, the progress in polymer science has added several plastics into the market, emphasizing the need for a comprehensive tagging system including factors like colour for better material recycling. Similarly, certifications and permits associated with labelling should be updated to reflect modern scientific understanding and findings. Additionally, eco-labels, such as those indicating biodegradability, plastic-free, or eco-friendly, issued by third-party certifiers assist the plastic recycling ecosystem. For example, the label (green dot) introduced under the producer responsibility for plastic packaging products in Germany boosted the recovery of recyclable plastics^6^. In contrast, the positive impact can remain unrealized when the product features generic and self-declared misleading claims to greenwash and confuse consumer decision-making^38^. For example, “100% Recyclable” (Coca-Cola and Nestle)^39^, “Degradable” (Coco Thumb), and “Microplastics Free” (Wital tea) without scientific merit to attract green purchases amplify the gravity of the situation.

## Environmental impacts of recycling

The use of plastic is anticipated to triple by 2060 compared to 2019, driven by the expanding global economy; however, the recycling rate may double during this period, creating a significant unintended environmental leakage^2,40^. Until now, the environment has been housing multiple layers of first-generation nonbiodegradable plastics that have transgressed different compartments^4^, which may unfold as a catastrophic environmental challenge. It is estimated that 19–23 Mt of plastic waste generated globally in 2016 entered aquatic ecosystems, but could reach up to 53 Mt annually by 2030^3^. Legacy plastic pollution is not just limited to marine and aquatic ecosystems. Due to the widespread use of plastics in agriculture and their limited recyclability, an estimated 12.5 Mt of plastics accumulate in agricultural soils annually^1,41^. Additionally, recycling alone cannot reverse the damage incurred due to the leakage of plastics already in the environment^21,35^.

Plastic recycling encompasses both positive and negative aspects, warranting a comprehensive evaluation to balance environmental benefits and burdens. Recycling plastic waste significantly reduces fossil fuel utilization, power consumption, and landfilling^30,42^. The ripple effect is a decline in the emission of greenhouse gases, thus lowering the carbon footprints while contributing to the global economy and direct jobs. In fact, it is emphasized that reprocessing 1 ton of plastic can save up to approximately 130 million kilojoules of energy^24^. A life cycle assessment (LCA) conducted on the environmental impact of 1) recycling plastic waste compared to alternative approaches and 2) application of secondary products instead of virgin materials marks a positive step toward climate control^30^. Similarly, several other LCA studies have confirmed the superiority of plastics as material over their alternative option such as aluminium bottles, paper, and cotton bags^43,44^. However, a notable limitation in several standard LCA methodologies lies in omitting a crucial factor—the long-term fate of chemicals and particulates released during EoL plastic^1,45^. The disadvantage of existing short-term LCAs in disregarding the consequences of chemical and particulate releases raises concerns about the overall efficacy of plastics and recycling as a solution to plastic pollution. This gap in evaluating the true ecological footprint of virgin and recyclate plastics (i.e., raw materials transported to a waste recycling facility for processing into a new materials or products) may result in unintended environmental and health costs.

Recycling facilities have been identified as potential hotspots and contributors of toxic and hazardous waste, however, there is limited attention to chemical or particle release from plastic recycling facilities. Despite the current and emerging technologies to recycle plastic waste, non-recoverable tiny plastic particles (microplastics) cannot be addressed with existing collection methods due to their exceptionally small size. Further, the size reduction and washing during mechanical recycling facilities tend to release significant microplastics into the environment^46^. About 13% of plastics infiltrate water or air as microplastics from recycling facilities in the UK^47^. A study on PET recycling facilities reveals microplastic releases range from approximately 23–1836 mg/L in wastewater that is distributed in the effluent (8–83 mg/L) and the sludge (52,166–68,866 mg/L) as it leaves the facility^48^. Microplastics generated during the recycling process are governed by the properties of plastics (polymer type or hardness) and environmental exposure^46^. Ideally, plastic recycling facilities are equipped with filters to prevent and mitigate environmental contamination, but it partially mitigates microplastic release and is not a comprehensive solution^47^. Additionally, the leaching of harmful plastic chemicals during and after recycling also poses a significant threat^29^. Recycled plastics exhibit higher levels of hazardous chemicals such as brominated flame retardants as legacy contaminants. The contamination not only hinders the wide application, it also poses health risks for workers and end-users^12^. With this, it is imperative to produce toxic chemical-free material through controls over what is being recycled and standards for recycled plastics and their usability in different sectors.

While chemical recycling can produce food-grade plastics and has been heralded to fix plastics recycling, it is financially risky and can have far-reaching environmental implications compared to virgin plastics production^8,21^. The damage to the environment through chemical recycling in terms of emissions, energy consumption, and water utilization surpasses those used in other technologies^49^. Meanwhile, mechanical recycling is believed to exhibit a lower overall impact on climate change than chemical recycling and energy recovery, which contributes to greenhouse gas emissions and photochemical ozone formation^42^. To address these concerns effectively, the transport and sorting of waste should be confined within closed spaces, filters should be installed and wastewater should be treated to prohibit the release of plastics and associated chemicals into the environment^36,50^. Despite an apparent increase in the plastic recycling rate, lower-grade polymers with a limited lifespan are eventually disposed of as waste, thus challenging the circular economy of plastics and environmental sustainability.

Inefficient waste collection, coupled with the necessity for sorting before recycling, requires transportation to dedicated waste handling facilities leading to inadvertent loss and an escalation in carbon footprints. However, the global plastic waste trade is built on the premise of exporting for recycling, often to lower-income countries^51^. Countries are also fraught with widespread environmental impacts and incredibly low recycling rates if accurately reported^52^. Further, regional policies have far-reaching effects on global plastics recycling dynamics. Until 2018, China had been the reprocessing house for more than 50% of PET bottles^53^, but the recent ban on foreign waste imports, including plastics, has left world recycling facilities scrambling^54^. High-income countries began exporting plastic waste to other low-income countries, particularly those in the global south^51,55^. Many of these low-income countries have become disproportionally impacted by plastic pollution due to overwhelming imports of plastic waste (for so-called “recycling”), as part of the global plastic waste trade^52^. These countries lack adequate recycling facilities, which has led to excessive open-dumping or burning of plastic waste, including waste-to-energy incineration^35,51^. Imported plastic, often of low quality, contaminated, or mislabelled, is diverted to landfilling and incineration, each contributing to negative environmental impacts. The other example of change in plastic waste dynamics includes the largest exporter of plastics (i.e., Japan), which saw a surge in reprocessing, while the use of virgin plastics increased in China which further increased the carbon footprint following the import ban^56^.

## Achieving plastic circularity and plastic recycling in the Global Plastics Treaty

Currently, we are in the midst of a global plastic pollution problem driven by unsustainable plastic production and plastic consumption^20^. The plastics industry narrative has previously been framed around the unique recyclability of many plastic polymers, but the reality is that plastics have been grossly mismanaged^3,57^. While recycling plays a role in managing plastic waste, doubts linger if it is a holistic solution^21^. The combination of poor polymer and product design, the nature of mixed waste generated, inadequate and wide variations of waste management infrastructure, poor quality of post-recycling products, demand-supply gaps, and environmental, economic, and social impacts have resulted in unsustainable plastic waste generation^7,19^. With technological limitations and substandard industrial compliance, plastic recycling is not working. Globally, the recycling rates for plastic are paling in comparison to paper and metals, with a high recycling rate of aluminium at 76%^58^. Even if plastics are recycled, the environmental impacts are startling, particularly with chemical recycling^42^.

Addressing the challenge of reducing global plastic production is complex, particularly given the disparity in plastic consumption between developing and developed economies. With almost 4 billion people residing in developing countries utilizing considerably less plastic than their counterparts in developed nations, there exists a growing trend towards increased production and usage in these regions. Further, the global trade in plastic waste often involves shipping to countries with lower processing costs. The extended producer responsibility (EPR) schemes have the potential to internalize the environmental costs of production and waste management, providing incentives to reduce the use of virgin plastics and improve the quality of recyclables^59^.

The transformative shift to global plastic sustainability demands a 50% reduction in future plastic demand, coupled with phasing out of fossil-derived plastics, a remarkable 95% recycling rate for retrievable plastics, and a transition to renewable energy sources to establish a sustainable circular plastics economy^60^. Although current technology for plastic recycling is yet not circular, robust steps in tandem with changing regulations and research efforts are needed to encourage a decline in the impact of plastics. The time lag to achieve a complete closed-loop recycling for all plastic produced accentuates the need to cap production and explore design-for-recyclability, extending beyond mere reducing and reusing these materials. Bridging the gap between escalating plastic production and effective recycling demands substantial immediate investment in research and infrastructure to maintain the plastic waste within the value chain without resorting to down-cycling or disposal.

Achieving sustainability and a circular economy requires recognizing the importance of methods beyond recycling, including product design, alternative materials, phasing out problematic plastics, curbing the consumption of virgin plastic materials, and adopting reduction and reuse strategies^23^ (Fig. 1). The paradigm shift necessitates a decoupling from fossil fuel reliance and embracing recycled and biobased feedstock, towards CO_2_ emission neutrality. Importantly, the focus extends to EoL considerations, where plastics should either be efficiently collected and economically recycled or designed to be completely biodegradable if dispersion is unavoidable^61,62^. Crucially, future polymer designs should not only meet traditional performance and cost but also incorporate safe and sustainable-by-design principles. A simplified plastic with a design-for-recyclability along with controlled chemicals, labels, and adhesive in finished products has the potential to encourage recycling rate^11,50^. Embracing a mono-material approach in product design, where single polymers are utilized without compromising performance, and innovative solutions such as debonding-on-demand techniques offer pathways to address the challenges posed by multilayer plastics products^61^. Additionally, establishing standards and global policies is crucial to capping plastic production and curbing the continuous flow of plastic waste into the environment^63^.

The reaction to the looming global threat of irreversible plastic pollution is through decreasing plastic emissions^64^. Life cycle analyses indicate net-zero emission plastics are achievable using current technology, through a synergistic approach that integrates biomass, CO_2_ utilization, and attains a 70% effective recycling rate, which significantly reduces energy use and operational costs^65^. Further, addressing the global plastic waste crisis requires the implementation of internationally coordinated waste management strategies^64^. Countries are implementing economic instruments to stimulate plastic recycling via different methods under the polluter-pays principle including EPR^66^, deposit-refund schemes (DRS), tax on virgin plastics, landfill and incineration taxes, and pay-as-you-throw schemes^67,68^. For instance, DRS, a lucrative refund incentive once applied to glass bottles, successfully promotes collection and reduces plastic littering. DRS accumulates less contaminated plastics over the traditional single-stream recycling process. The scheme has incentivized as high as 95% of plastic bottle recycling in Norway whereas Ecuador reported an 80% collection of PET bottles in 2012 as compared to 30% in 2011^69^. Similarly, in 2019 plastic collection under DRS has increased in different countries including Denmark (94%), Croatia (89%), Estonia (87%), and Finland (90%)^69^.

The challenge of EoL plastic has been recognized by the international community with 175 United Nations member countries agreeing to eliminate plastic pollution with a legally binding plastic treaty instrument^70^. The international community with the ongoing Plastics Treaty negotiations have already established a zero draft document and an updated revised zero draft document, which includes elements to address inadequacies of current plastic recycling^50^. Those include primary plastic polymers, chemicals, and polymers of concern when recycling complex mixtures of plastic waste^71^. Additionally, problematic and avoidable single-use plastic products will be included in the Global Plastics Treaty as these are invariably difficult or impossible to recycle and should be phased out or replaced with sustainable alternatives^72–74^. Sustainable product design, performance, and practices such as reduction, reuse, refill, and repair will be emphasized.

Another important element of the Global Plastics Treaty includes the use of increased recycled plastic contents amidst the challenge of rising global plastic production, largely from virgin plastics^21^. To increase recycled plastic contents as part of the Global Plastics Treaty, governments could implement economic policy instruments to incentivise the price of recycled plastics compared to virgin plastics. For example, industries utilizing recycled plastics could be offered lower corporate taxes, whereas industries using virgin plastics would incur penalties (higher corporate taxes). The transition to a circular economy needs to reduce resource consumption and plastic pollution by moving away from the current linear economic model of plastic production^63^. Only focus on improved recycling and improvements in waste management facilities will promote increased production of waste as it will not cap production and will effectively lock-in the global community to business as usual.

Finally, the Global Plastics Treaty will also include elements of EPR, emissions and releases of plastic through its entire life cycle, transformational improvements to waste management, as well as a just transition for waste pickers who play a major role in driving the informal recycling sector in many jurisdictions. Overall, it will offer opportunities to improve plastic recycling and eliminate harmful chemicals used in plastic production, manufacture, and packaging.

## Concluding remarks

An immense variety of plastic products comprising a complex mixture are used in every aspect of modern society. However, the sustainability of these invaluable materials has largely been ignored. A staggering 91% of plastic meets an alternate fate than recycling. To improve the sustainability of plastic recycling we need a coordinated global panacea of solutions, as there is no one silver bullet to solve the pervasive plastic pollution problem. Emerging recycling technologies will help contribute to the panacea of solutions, but without global coordination, such as the Global Plastics Treaty, they alone will not address the plastic pollution crisis until it is controlled at the source with plastic production caps. Under the Global Plastics Treaty, United Nations member countries could consider adjusting the international price of virgin plastics to reflect the true environmental and economic costs of plastic pollution on ecological and human health. Reducing global virgin plastic production and overall consumption will help the implementation of an effective Global Plastics Treaty that will comprise comprehensive elements to reduce plastic pollution and increase plastic recycling to achieve a circular economy.

## Data Availability

No datasets were generated or analysed during the current study.
